# Impacts of Birth Weight on Plasma, Liver and Skeletal Muscle Neutral Amino Acid Profiles and Intestinal Amino Acid Transporters in Suckling Huanjiang Mini-Piglets

**DOI:** 10.1371/journal.pone.0050921

**Published:** 2012-12-07

**Authors:** Huansheng Yang, Dezhi Fu, Hua Shao, Xiangfeng Kong, Wence Wang, Xiaojian Yang, Charles M. Nyachoti, Yulong Yin

**Affiliations:** 1 Key Laboratory of Agro-ecological Processes in Subtropical Region and Research Center for Healthy Breeding of Livestock and Poultry, Institute of Subtropical Agriculture, Chinese Academy of Sciences, Changsha, Hunan, People’s Republic of China; 2 Huanjiang Observation and Research Station for Karst Ecosystem, Chinese Academy of Sciences, Huanjiang, Guangxi, People’s Republic of China; 3 Department of Animal and Poultry Science, University of Guelph, Guelph, Ontario, Canada; 4 Department of Animal Science, University of Manitoba, Winnipeg, Manitoba, Canada; 5 Ottawa Hospital Research Institute, University of Ottawa, Ottawa, Ontario, Canada; 6 Graduate University of Chinese Academy of Sciences, Beijing, People’s Republic of China; National Cancer Institute, United States of America

## Abstract

Genetic selection strategies towards increased prolificacy have resulted in more and more increased littler size and incidences of impaired fetal development. Low birth weight (LBW) piglets, with long-term alterations in structure, physiology and metabolism, have lower survival rates and poor growth performance. The aim of the study was to compare the plasma, liver and skeletal muscle contents of neutral amino acids (NAA) and the intestinal expression of NAA transporters between LBW and high birth weight (HBW) suckling Huanjiang mini-piglets. Forty piglets with either LBW or HBW (20 piglets per group) were sampled on day 0, 7, 14 and 21 of age to give 5 observations per day per group. The contents of NAA in plasma, liver and skeletal muscle were measured, and jejunal expression of transporters for NAA, including *Slc6a19* (B^0^AT1) and *Slc1a5* (ASCT2), were determined by real-time RT-PCR and Western Blot, respectively. Results showed that the suckling piglets with LBW had higher contents of Thr, Ser, Gly, Ala, Val, Met, Ile, Leu, Tyr, Phe and Pro in liver, and Gly in skeletal muscle, whereas lower contents of Met, Ser and Ala in plasma when compared with the HBW littermates. Consistent with the content differences in plasma NAA, the jejunal expression profiles of both *Slc6a19* (B^0^AT1) and *Slc1a5* (ASCT2) in the LBW piglets were lower in compared with the HBW littermates during the early suckling period. These findings suggested that intestinal dysfunction in the LBW piglets may be one of the reasons in altered physiology and metabolism states of other organs, which result in lower survival and growth rate.

## Introduction

Birth weight (BW) and its variation within a litter is an important economic trait in animal production, because low BW (LBW) in animal correlates with lower survival rates, poor growth performance and sub-optimal carcass quality [1]–[4]. However, genetic selection for large litter during the last decades has resulted in lower mean BW [5], [6]. Moreover, in some animals, such as pigs, there is a 2- to 3-fold variation in BW among littermates from normally fed sows because of differences in placental size and functional capacity [7]. Low BW results from intrauterine growth retardation (IUGR) during gestation [4], which occurs in animals as a consequence of fetal adaptation to adverse fetal environments, leading to molecular and physiological adaptive changes [8]. Although this fetal adaptation allows fetal survival, it also results in permanent alterations in structure, physiology and metabolism [9].

Intestines, muscle and liver are major organs involved in digestion, absorption and metabolism of dietary nutrients [10]. The intestinal function was impaired in LBW piglets at birth with lower lactase and aminopeptidase N peak, and reduced relative weight of the pancreas [11], [12]. Pigs with LBW exhibited a lower carcass quality in terms of lower lean mass and higher fat deposition at market weight [4]. These body composition alterations might be the consequences of reduced mRNA translation and energy sensing, and impaired oxidative phosphorylation in skeletal muscle [13], [14]. Liver plays a major role in the nutrient metabolism, such as glucose, lipids and amino acids [15], [16]. The brain to liver ratio was increased in LBW fetal. In other words, the LBW fetal liver is smaller relative to the brain as brain weight is poorly affected by BW [12], [14]. These alterations may be associated with dysfunction of absorption and metabolism of nutrients, such as amino acids (AA).

Neutral amino acids (NAA) are not only building blocks for tissue proteins but also regulators of hormone secretion, cell signaling molecules, and precursors for the synthesis of non-protein substances with biological importance. Obviously, NAA play irreplaceable roles in maintaining normal physiological function, growth and development of living organism. NAA in the intestine are mainly transported by B^0^AT1 and ASCT2, both of which are expressed in the jejunum, the major site of AA absorption [17]. B^0^AT1 transports all the NAA and most of the essential AA, and ASCT2 mediates transport of NAA with the exception of aromatic AA with high affinity.

Huanjiang mini-pig is a well-known indigenous breed which is mainly distributed in the southern China [18]. Because of its small size and similar anatomical, physiological and metabolic characteristics to human, it is increasingly viewed as a suitable experimental model [19]. Considering that LBW is accompanied with structure, physiology and metabolism alterations of many organs after birth, we hypothesized that LBW may be associated with alterations in the absorption of NAA, which may result in their compositional changes in key tissues. In order to test this hypothesis, we examined the jejunal expression of B^0^AT1 and ASCT2 and NAA contents in plasma, skeletal muscle and liver of suckling piglets with LBW or HBW.

## Materials and Methods

### Animals and Sample Collection

Twenty littermates of suckling Huanjiang mini-piglets were used and nursed by primiparous gilts in the present study. The gilts were individually housed and fed a maize- and soybean meal-based diet and housed in the same pigsty [20]. On days 0, 7, 14 and 21 of age, five littermates were chosen and two piglets from per littermate (one with the largest BW and another with the lowest BW) were sampled, respectively. Piglets were individually weighed immediately before feeding. Blood samples (about 5 ml from each piglet) were collected into 10-mL heparin-coated tubes and centrifuged at 3,000×*g* and 4°C for 10 min. Then, the supernatants (plasma) were stored at −20°C until required for analysis of AA content. Immediately after blood sampling, piglets held under general anaesthesia and then killed by an intravenous injection of the 4% sodium pentobarbital solution (40 mg/kg body weight) [21]. Samples of proximal jejunum (after cleaned by ice-cold phosphate-buffered saline), longissimus dorsi muscle, and liver were collected and immediately frozen in liquid nitrogen and then stored at −70°C until analysis. All the experimental procedures used in this study were approved by the Animal Care and Use Committee of Chinese Academy of Sciences [22]–[23].

### Determination of AA Contents in Plasma, Liver and Muscle

Plasma AA contents were determined as previously described [24]–[25]. In brief, 1 ml of the plasma sample and 2.5 ml of 7.5% trichloracetic acid solution were mixed thoroughly and centrifuged at 12,000×*g* and 4°C for 15 min. The supernatant fluid was collected for analysis of AA by an ion-exchange AA analyzer (Tokyo, Japan).

To measure the contents of AA in the muscle and liver, about 0.1 g freeze-dried muscle or liver tissue was ground and hydrolyzed in 10 mL of 6 mol/L HCl at 110°C for 24 h. The solution was then adjusted to the volume of 100 mL and then a 1 mL of the settled solution was used for further analysis after a 10-fold dilution. Plasma samples were filtered through a 0.45 µm membrane before analysis [26].

### RNA Extraction and cDNA Synthesis

Approximately 100 mg of tissue from each jejunal sample was pulverized in liquid nitrogen [27]. Total RNA was isolated from homogenate using the TRIZOL reagent (Invitrogen, CA, USA). The RNA integrity was checked by 1% agarose gel electrophoresis, stained with 10 µg/mL ethidium bromide. The quantity of RNA were determined by ultraviolet spectroscopy using a NanoDrop® ND-1000 (Thermo Fisher Scientific, DE, USA). RNA was treated with DNase I (Invitrogen, CA, USA) according to the manufacturer’s instructions before reverse transcription and polymerase chain reaction (PCR). Synthesis of the first strand cDNA was performed with Oligo (dT) 20 and Superscript II reverse-transcriptase (Invitrogen, CA, USA).

### Relative Quantification of Gene Expression of Slc6a19 and Slc1a5

Primers for the selected genes (Table 1) were designed using Oligo 6.0 software. Real-time quantitative PCR analyses were performed with 5 ng of reverse-transcribed RNA and both sense and anti-sense primers in a final volume of 10 µL using SYBR Green I as a PCR core reagent (TaKaRa, Dalian, China). After a pre-denaturation program (10 s at 95°C), forty cycles of amplification were conducted with each cycle consisting of 95°C for 10 s, 60°C for 20 s, and following by a melting curve program (60 to 99°C with heating rate of 0.1°C/s and fluorescence measurement). The amplification of GAPDH was used for each sample to normalize the expression of the selected genes. The relative expression ratio (R) of mRNA was calculated by R = 2^(Ct GAPDH − Ct test)^. Real-time reverse-transcription PCR efficiencies were acquired by the amplification of dilution series of cDNA according to the equation 10^(−1/slope)^ and consistent between target mRNA and *GAPDH* mRNA. Negative controls were performed in which cDNA was substituted for water.

**Table 1:** Primers used for real-time PCR.

Genes	Primers	Sequences(5′-3′)	Size(bp)	TA^1^(°C)
*Slc1a5*	Forward	GATTGTGGAGATGGAGGATGTGG	128	58
	Reverse	TGCGAGTGAAGAGGAAGTAGATGA GA		
*Slc6a19*	Forward	TCTGTCCACAACAACTGCGAG	206	57
	Reverse	CAGCGAAGTTCTCCTGCGTC		
*GAPDH*	Forward	AAGGAGTAAGAGCCCCTGGA	140	60
	Reverse	TCTGGGATGGAAACTGGAA		

1 TA, Annealing temperature.

### Determination of Protein Quantity of ASCT2 and B0AT1

The frozen jejunum samples were powdered under liquid nitrogen, and lysed in RIPA buffer (150 mM NaCl, 1% Triton X-100, 0.5% sodium deoxycholate, 0.1% SDS, 50 mM Tris-HCl at pH 7.4, plus a protease inhibitor cocktail purchased from Roche, Shanghai, China). After centrifugation at 10,000×*g* and 4°C for 10 min, protein concentration in the supernatant fluid was determined using the Bicinchoninic Acid assay (Beyotime Biotechnology, Haimen, China). All samples were adjusted to an equal protein concentration and then diluted with 2×loading buffer (0.63 ml of 0.5 M Tris-HCl (pH 6.8), 0.42 ml 75% glycerol, 0.125 g sodium dodecyl sulfate (SDS), 0.25 ml β-mercaptoethanol, 0.2 ml 0.05% solution of bromphenol blue, and 1 ml water) to a final volume of 2.5 ml and heated in boiling water for 5 min. After cooled on ice, the solution was used for Western blot analysis.

Same amounts of sample aliquots (20 µg protein) were subjected to 10% SDS-PAGE (10% gradient gel) and were transferred to PVDF membranes (Millipore, MA, USA) overnight at 12 V using the Bio-Rad Transblot apparatus (CA, USA). The membranes were blocked in 5% fat-free milk in Tris-Tween buffered saline (TTBS; 20 mM Tris/150 mM NaCl, pH 7.5, and 0.1% Tween-20) for 3 h and then incubated with ASCT2, B0AT1 or β-actin antibody (Table 2) at 4^o^C overnight with gentle rocking. After washing three times with TTBS, the membranes were incubated at room temperature for 2 h with horseradish peroxidase-linked secondary antibodies (Santa Cruz, CA, USA). The secondary antibody were used at dilutions of 1∶3,000. Finally, the membranes were washed with TTBS, followed by development using Supersignal West Dura Extended Duration Substrate according to the manufacturer’s instructions (Pierce, Rockford, IL). The images were detected on chemiluminescence (Applygen Technologies Inc., Beijing, China). Multiple exposures of each Western blot were performed to ensure linearity of chemiluminescence signals. Western blots were quantified by measuring the intensity of correctly sized bands using AlphaImager 2200 software (Alpha Innotech Corporation, CA, USA). The ratio of intensities of a studied protein band and housekeeping protein band was calculated for each filter and the ratios from different Western blot filters were used for analyzing the abundances of studied proteins.

**Table 2:** Antibodies and dilution used for Western blot analyses.

Antibody	Company	Catalog Number	Dilution
ASCT2	Santa Cruz, CA, USA	sc130963	1∶500
B^0^AT1	Santa Cruz, CA, USA	sc160811	1∶1000
β-actin	Santa Cruz, CA, USA	sc47778	1∶1000

### Statistical Analysis

The data were analyzed by a mixed-effects model using the SAS version 9.2. The statistical model used included the main effects of BW size, age, and their interactions, age entered the model as a repeated measure with sow within BW class as a subject, and the individual sow was served as a random effect. Probability values <0.05 were taken to indicate statistical significance.

## Results

### Body Weight and Plasma Contents of NAA in Huanjiang Mini-piglets with LBW or HBW

The LBW piglets showed lower body weight than the HBW pigs during the whole suckling period (Table 3). Compared with the HBW piglets, LBW piglets had a lower (*P*<0.05) plasma content of Met on day 0 of age, as well as of Ser and Ala on day 7 of age. No significant differences in plasma contents of other NAA between HBW and LBW piglets were noted from days 0 to 21 of age (Table 4). The plasma content of Ser, Cys and Met in piglet was decreased (*P*<0.05) with the increase of age. Age×BW interaction effects were noted for plasma content of Ser and Met in suckling Huanjiang mini-piglets (*P*<0.05; Table 4). No interaction effects of age×BW were observed on other detected NAA.

**Table 3:** Body weight as a function of age for piglets with HBW and LBW.

Day ofage	0	7	14	21
N^1^	5	5	5	5
HBW^2^ (g)	654.1±32.71	1387.5±70.29	1937.8±97.22	2624.4±195.59
LBW^3^ (g)	370.2±19.97*	771.7±53.55 *	1064.1±81.09 *	1320.7±94.08 *

Data are expressed as mean ± SD. Values in a row with * differ (*P*<0.05).

1 N, number of piglets per birth weight.

2 HBW, high birth weight;

3 LBW, low birth weight.

**Table 4:** Plasma contents (µmol/L) of neutral amino acids in Huanjiang mini-piglets with HBW^1^ and LBW^2^.

Item	Day of age									*P*-value		
	0		7		14		21					
	HBW	LBW	HBW	LBW	HBW	LBW	HBW	LBW	SEM	Age	BW	Age×BW
Thr	757.9	582.2	813.5	642.4	358.8	395.4	380.6	377.8	97.69	0.330	0.362	0.829
Ser	347.8^ bc^	279.7^ c^	474.1^ a^	281^ c^	198.1^d^	190.5^ d^	167.5^ d^	165.5^d^	36.91	0.001	0.038	0.046
Gly	624.4	539.5	1127.8	776.9	739.4	688.3	782.9	731.2	33.41	0.136	0.113	0.660
Ala	446.7	421.7	674.1^ a^	490^ b^	454.9	410.9	452.2	465.0	24.43	0.693	0.009	0.776
Cys	151.9	138.1	148.9	147.1	137.9	141.7	153.8	145.6	7.13	<0.001	0.276	0.393
Val	406.0	370.6	355.4	359.9	250.3	284.2	303.3	297.7	16.03	0.079	0.415	0.568
Met	140.7^ a^	96.4^ b^	81.8^ bc^	62.2^ cd^	38.2^ d^	45.8^ d^	49.0^ d^	46.4^ d^	7.74	0.009	0.015	0.037
Ile	78.2	54.1	115.9	140.3	74.6	88.4	123.9	99.8	7.54	0.144	0.836	0.385
Leu	239.8	238.8	217.0	216.8	150.2	170.3	193.4	179.1	7.49	0.484	0.504	0.736
Tyr	114.0	110.8	111.0	122.6	77.0	78.3	81.8	81.4	5.13	0.712	0.827	0.943
Phe	171.2	151.5	151.4	139.4	113.4	112.7	118.3	130.3	8.47	0.124	0.266	0.449
Pro	506.8	495.1	465.6	433.5	484.5	457.9	431.9	449.1	12.39	0.267	0.318	0.770

a-d Values within a row without a common superscript letter differ (*P*<0.05).

1 HBW, high birth weight;

2 LBW, low birth weight.

### Liver Contents of NAA in Huanjiang Mini-piglets with LBW or HBW

Liver contents of 12 NAA of piglets with LBW or HBW from days 0 to 21 of age are shown in Table 5. Compared with the HBW piglets, the liver contents of 12 measured NAA, excepting Cys, in the LBW piglets were higher (*P*<0.05) on day 0 of age, whereas LBW piglets had lower content of Cys on day 14 of age. No significant differences in liver contents of NAA between LBW and HBW piglets was observed on days 7 and 21 of age. The liver content of all measured NAA, excepting Cys, in piglets was increased (*P*<0.001) from days 0 to 21 of age. There were interaction effects between age and BW on liver content of all measured NAA in suckling Huanjiang mini-piglets (*P*<0.05).

**Table 5:** Liver contents (%) of neutral amino acids in Huanjiang mini-piglets with HBW^1^ and LBW 2.

Item	Day of age									*P*-value		
	0		7		14		21					
	HBW	LBW	HBW	LBW	HBW	LBW	HBW	LBW	SEM	Age	BW	Age×BW
Thr	0.30 ^e^	0.45^ d^	0.54^ abcd^	0.48^ cd^	0.58^ab^	0.57^ bc^	0.63^ a^	0.60^ ab^	0.042	<0.001	0.562	0.007
Ser	0.30 ^e^	0.44^ d^	0.52^ abc^	0.46 ^cd^	0.55^ ab^	0.53^ bc^	0.59^ a^	0.56^ ab^	0.038	<0.001	0.727	0.004
Gly	0.36^ c^	0.57^ b^	0.64^ ab^	0.56^ b^	0.69^ a^	0.67^ ab^	0.74^ a^	0.71^ a^	0.055	<0.001	0.513	0.003
Ala	0.37^ c^	0.57^ b^	0.68^ ab^	0.58^ b^	0.73^ a^	0.71^ a^	0.80^ a^	0.75^ a^	0.057	<0.001	0.694	0.004
Cys	0.42 ^bc^	0.45^ ab^	0.43^ b^	0.43^ b^	0.46^ a^	0.44^ b^	0.41^ c^	0.43^ bc^	0.010	0.009	0.315	0.014
Val	0.48^ d^	0.67^ c^	0.82^ ab^	0.71^ bc^	0.86^ a^	0.83^ ab^	0.89^ a^	0.85^ a^	0.062	<0.001	0.994	0.004
Met	0.21^ d^	0.25^ c^	0.27^ bc^	0.25^ c^	0.28^ b^	0.28^ b^	0.32^ a^	0.31^ ab^	0.018	<0.001	0.818	0.045
Ile	0.33^ d^	0.49^ c^	0.60^ abc^	0.53^ bc^	0.65^ a^	0.64^ a^	0.69^ a^	0.66^ a^	0.044	<0.001	0.647	0.004
Leu	0.57^ d^	0.87^ c^	1.08^ bc^	0.94^ c^	1.16^ b^	1.13^ b^	1.29^ a^	1.19^ ab^	0.087	<0.001	0.833	0.004
Tyr	0.32^ e^	0.40^ d^	0.47^ bc^	0.44^ cd^	0.50^ ab^	0.49^ bc^	0.54^ a^	0.52^ ab^	0.026	<0.001	0.836	0.031
Phe	0.58 ^e^	0.69^ d^	0.79 ^bc^	0.76^ cd^	0.81 ^bc^	0.87 ^b^	1.02^ a^	0.95^ a^	0.047	<0.001	0.479	0.041
Pro	0.48^ d^	0.76^ c^	0.92^ abc^	0.80 ^bc^	1.05^ a^	0.96^ ab^	1.03^ a^	1.00^ a^	0.084	<0.001	0.789	0.009

a-e Values within a row without a common superscript letter differ (*P*<0.05).

1 HBW, high birth weight;

2 LBW, low birth weight.

### Muscle Contents of NAA in Huanjiang Mini-piglets with LBW or HBW

The muscle contents of 12 NAA in piglets with LBW or HBW from days 0 to 21 of age was examined. Compared with the HBW piglets, the LBW piglets had a higher (*P*<0.05) muscle contents of Gly on day 0 of age. No significant differences in NAA content between LBW and HBW piglets were noted from days 7 to 21 of age (Table 6). The muscle content of all measured NAA, excepting Gly and Pro, in piglets was increased (*P*<0.001) from days 0 to 21 of age. An age×BW interaction effect was noted for muscle content of Gly in suckling Huanjiang mini-piglets (*P*<0.05; Table 6). No interaction effects of age×BW were observed on other detected NAA.

**Table 6:** Skeletal muscle content (%) of neutral amino acids in Huanjiang mini- piglets with HBW^1^ and LBW 2.

Item	Day of age									*P*-value		
	0		7		14		21					
	HBW	LBW	HBW	LBW	HBW	LBW	HBW	LBW	SEM	Age	BW	Age×BW
Thr	0.44	0.43	0.63	0.64	0.69	0.62	0.71	0.69	0.049	<0.001	0.247	0.565
Ser	0.41	0.41	0.56	0.56	0.58	0.50	0.59	0.59	0.049	<0.001	0.412	0.653
Gly	0.63 ^b^	0.80^ a^	0.72^ ab^	0.74^ ab^	0.71^ ab^	0.70 ^ab^	0.71^ ab^	0.71^ ab^	0.045	0.889	0.037	0.018
Ala	0.60	0.63	0.80	0.80	0.85	0.85	0.87	0.84	0.067	0.001	0.954	0.989
Cys	0.40	0.40	0.43	0.41	0.44	0.46	0.43	0.43	0.012	<0.001	0.238	0.474
Val	0.59	0.55	0.80	0.79	0.84	0.78	0.86	0.83	0.055	0.001	0.229	0.883
Met	0.25	0.24	0.32	0.31	0.36	0.36	0.36	0.34	0.023	<0.001	0.392	0.987
Ile	0.47	0.44	0.66	0.67	0.75	0.67	0.76	0.73	0.048	<0.001	0.095	0.205
Leu	0.77	0.73	1.09	1.10	1.21	1.13	1.24	1.21	0.090	0.001	0.399	0.687
Tyr	0.39	0.35	0.50	0.51	0.49	0.56	0.60	0.56	0.052	<0.001	0.994	0.463
Phe	0.52	0.50	0.64	0.66	0.70	0.68	0.71	0.70	0.037	<0.001	0.477	0.290
Pro	0.87	1.02	1.06	1.06	1.05	1.06	1.01	1.01	0.046	0.078	0.447	0.206

a,b Values within a row without a common superscript letter differ (*P*<0.05).

1 HBW, high birth weight;

2 LBW, low birth weight.

### Expression Profiles of Jejunal Slc6a19 (B0AT1) and Slc1a5 (ASCT2) in Huanjiang Mini-piglets with LBW or HBW

**Figure 1:**
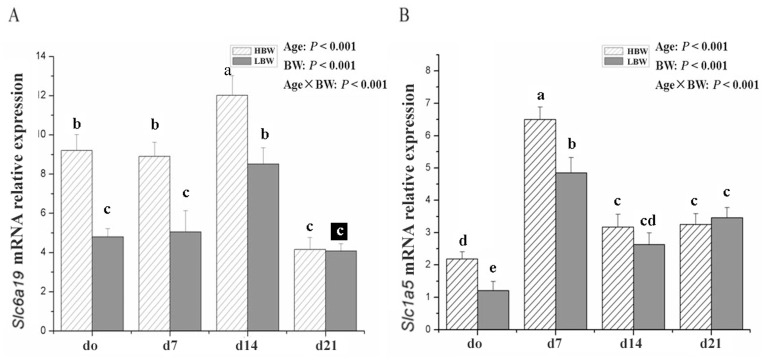
mRNA levels of jejunum *Slc6a19* (A) and *Slc1a5* (B) in sucking Huanjiang mini-piglets with HBW^1^ or LBW^2^ from days 0 to 21 of age. Samples were obtained from Huanjiang mini-pigs on Days 0, 7, 14 and 21 of age, respectively. The mRNA expression levels of *Slc6a19* and *Slc1a5* were normalized using *GAPDH* as an inner control. Values without a common letter on bars differ (*P*<0.05). The *P* value showed the effects of age, BW, and age and BW interaction on mRNA expressiong of *Slc6a19* and *Slc1a5.* Data are expressed as mean ± SD, n = 5. ^1^HBW, high birth weight; ^2^LBW, low birth weight.

The mRNA expression levels of both *Slc6a19* and *Slc1a5* were changed with age (*P*<0.001). Compared with the HBW piglets, the mRNA expression level of *Slc6a19* in the LBW was lower (*P*<0.05) on days 0, 7 and 14 of age, as well as of *Slc1a5* on days 0 and 7 of age. The differences of mRNA expression levels of *Slc6a19* and *Slc1a5* between the LBW and HBW piglets declined gradually from days 0 to 21 of age. No differences in mRNA expression level of *Slc6a19* were observed on days 14 and 21 of age, as well as of *Slc1a5* on day 21 of age. Age×BW interaction effects were observed for both *Slc6a19* and *Slc1a5* mRNA expression (*P*<0.001; Fig. 1).

**Figure 2:**
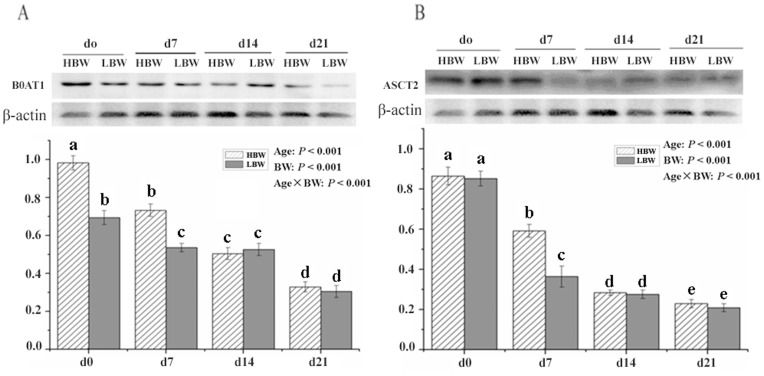
Protein abundances of jejunum B^0^AT1 (A) and ASCT2 (B) in sucking Huanjiang mini-piglets with HBW^1^ and LBW^2^ from days 0 to 21 of age. Samples were obtained from Huanjiang mini-pigs on Days 0, 7, 14 and 21 of age, respectively. The protein amounts of B0AT1 and ASCT2 were normalized using β-actin as an inner control. Values without a common letter on bars differ (*P*<0.05). The *P* value showed the effects of age, BW, and age and BW interaction on protein expressiong of B0AT1 and ASCT2. Data are expressed as mean ± SD, n = 5. ^1^HBW, high birth weight; ^2^LBW, low birth weight.

The protein abundances of both B^0^AT1 and ASCT2 were different from the mRNA expression levels. The protein expression of B^0^AT1 and ASCT2 was declined from days 0 to 21 of age (*P*<0.001). Compared with the HBW piglets, the LBW piglets had a lower (*P*<0.05) protein abundance of B^0^AT1 on days 0 and 7, as well as of ASCT2 on day 7 of age. No statistical differences in protein abundances of B^0^AT1 and ASCT2 were observed on days 14 and 21 of age. There were interaction between age and BW on both *Slc6a19* and *Slc1a5* protein expression(*P*<0.001; Fig. 2).

## Discussion

This study investigated the NAA contents of plasma, liver, and skeletal muscle, as well as jejunal expression profiles of their transporters in suckling Huanjiang mini-piglets with HBW or LBW. The novel and important findings from this study are that the LBW piglets had alterations in the contents of NAA in plasma (including Ser, Ala and Met), liver (including Thr, Ser, Gly, Ala, Cys, Val, Met, Ile, Leu, Tyr, Phe and Pro) and muscle (including Gly) during the early sucking period, which were associated with expression changes of their intestinal transporters at both mRNA and protein levels, with a lower expression level of *Slc6a19* (B0AT1) and *Slc1a5* (ASCT2) in the LBW piglets. There were age×BW interaction effects on plasma (including Ser and Met), liver (including Thr, Ser, Gly, Ala, Cys, Val, Met, Ile, Leu, Tyr, Phe and Pro) and muscle (including Gly) contents of NAA, as well as jejunal expression of *Slc6a19* (B0AT1) and *Slc1a5* (ASCT2) in suckling piglets. These findings suggested that the intestinal dysfunction of the LBW piglets may be one of the reasons for altered physiology and metabolism states of other organs, which result in lower survival and growth rate. The current study contributes further by providing new perspectives that might aid in the development of strategies to ameliorate the disadvantages experienced by LBW pigs.

The growth and development processes of pigs involve not only changes of weight and shape, but also alterations of chemical composition and physiological functions. Other studies showed that the HBW piglets had less fat and protein and more water than their littermates by chemical analysis of the whole body at birth [3]. In addition, the LBW pigs exhibited less lean mass and more fat at market weight than the HBW pigs [4]. Here our data indicated that the LBW piglets had alterations in the contents of NAA in plasma, liver and skeletal muscle during the early sucking period, with lower contents in plasma and higher contents in liver and skeletal muscle of some NAA. Moreover, age×BW interaction effects were observed for plasma, liver and skeletal muscle contents of some NAA in sucking piglets. It has been reported that arterial concentration of Ile was significantly reduced in the IUGR sheep fetuses [14]. According to our results, there were no differences in the contents of Ile in plasma and muscle between the HBW and LBW piglets, but a higher content in liver of LBW piglets.

Amino acids and peptides are mainly absorbed by the enterocytes of the small intestine, with the proximal jejunum as the major absorption site of AA and peptides. However, other studies showed that, compared with the normal BW piglets, IUGR reduced the height of villi and the average number of villi per unit area, which resulted in a lower intestinal surface area for nutrient absorption [28]. Moreover, the LBW piglets’ small intestine exhibited signs of immaturity, which may reduce the digestive and absorptive capacities [12]. To test whether the alterations of NAA contents in plasma, liver and skeletal muscle were related to the absorption process, the mRNA expression levels and protein abundances of two major NAA transporters, including *Slc6a19* (B0AT1) and *Slc1a5* (ASCT2), were examined. Consistent with the content alterations of NAA, the expression levels of both *Slc6a19* (B0AT1) and *Slc1a5* (ASCT2) were changed at early suckling period. These findings suggested a relationship between intestine dysfunction and physiological change of other organs in the LBW piglets. Further work should be conducted to confirm this relationship.

Other studies demonstrated that LBW in piglets correlates with decreased survival rates [2], [5], [6]. Two-thirds of piglets with BW less than 0.8 kg died during suckling, the mortality for piglets with BW of 0.81 to 1.0 kg is 34% and less than 10% for piglets above 1.6 kg BW [6]. More than Seventy-five percent of post-natal deaths for LBW piglets occurred within the first week after birth. According to the results of the present study, LBW piglets had alterations in contents of some of NAA in plasma, liver and skeletal muscle, and lower jejunal expression of *Slc6a19* (B0AT1) and *Slc1a5* (ASCT2) during the first week after birth, which suggested that the intestinal dysfunction may be one of the reason for the high mortality of LBW piglets. Other studies showed that the differences in the intestinal shape and enzymatic functions between IUGR and normal BW piglets lessen with the increase of age [12], the alterations in NAA contents and their transporters between HBW and LBW piglets also faded out with increasing age. Moreover, the difference in mortality of HBW and LBW piglets was also disappeared as animals became older [6].

Pigs with LBW required a longer growing time to reach the same market weight than their HBW littermates [29]. A number of possible mechanisms underlying these differences are under discussion. Long-term modifications in the growth-regulating hormonal axes could be the reason for lower growth performances of LBW neonates. Indeed, low BW piglets had a lower circulating concentration of IGF-1 compared with their HBW littermates [30]. Another hypothesis is that the LBW piglets consumed less milk per suckling and compete less effectively for food than their HBW littermates [31]. It is also possible that LBW suffer long-term negative effects on the efficiency of feed utilization, since the intestine of LBW piglets not only exhibited morphological changes but also with physiological and functional alterations. The results of the present study demonstrated inhibition of expression of NAA transporters in the jejunum of LBW piglets during the early suckling period, which is in agreement with the hypothesis the lower growth performances of LBW piglets may be due to their inefficiency in using dietary nutrient. The low intestinal capacity for AA transport in LBW piglets’ intestine would further limit the development and growth of piglets with an already lower BW. Although the differences in plasma, liver and skeletal muscle NAA contents, and jejunal expression of transporters for NAA between LBW and HBW piglets was gradually disappeared during suckling, the difference in growth performance between LBW and HBW pigs was also disappeared after post-weaning period, and the highest difference in growth performance between LBW and HBW piglets was observed at suckling period [29].

In summary, our results showed that there were differences in the contents of some of NAA in plasma, liver and skeletal muscle of Huanjiang mini-piglets classified as LBW compared with those classified as HBW during the early suckling period. These changes were accompanied with the inhibition of the expression of NAA transporters in the small intestine. These findings suggested that dysfunctions in intestinal absorptive capacity for essential AA may be one of the factors involved in the negatively influence of low BW on mortality and growth performance in piglets. There is a need for further research to develop and test strategies for improving intestinal AA absorption, especially in low BW piglets.
